# The Effect of Pet Therapy and Artist Interactions on Quality of Life in Brain Tumor Patients: A Cross-Section of Art and Medicine in Dialog

**DOI:** 10.3390/bs8050043

**Published:** 2018-04-27

**Authors:** Stefan Petranek, Jennifer Pencek, Mahua Dey

**Affiliations:** 1 Herron School of Art & Design, Indiana University-Purdue University Indianapolis, Indianapolis, IN 46202, USA; spetrane@yahoo.com; 2 Department of Neurosurgery, Indiana University, Simon Cancer Center, Indianapolis, IN 46202, USA; jpencek@goodmancampbell.com

**Keywords:** brain tumor, malignant glioma, pet therapy, video art, photography, quality of life, cancer, artist, artistic engagement, art

## Abstract

With the evolution of modern medical treatment strategies, there also comes the realization that many times we reach a point where traditional goals of medical care, such as overall survival or disease-free survival, are not realistic goals for many patients facing devastating illnesses. One such disease is malignant primary brain tumors, known as malignant glioma (MG). With median survival of only 20.9 months following best available standard of care treatment strategies, including surgery, chemotherapy, radiation, and tumor treating fields, MG is one of the deadliest malignancies of the modern era. Along the course of treating patients with MG, clinicians often realize that traditional treatment therapies can at best provide incremental benefit of symptom management without any survival benefit. However, even in these difficult situations, it is possible to make significant positive changes in patients’ health-related quality of life (HRQoL) using creative, non-traditional interventions. In this paper, we describe the initial findings from our project that takes a unique approach to studying the intersections of clinical care and art by using pet therapy and art-making as interventions for patients diagnosed with brain tumors. Our preliminary findings suggest that pet therapy and the ability to reflect as well as speak about their journey through a life-altering disease significantly increases patients’ overall feeling of wellbeing and reduces anxiety about future uncertainty.

## 1. Introduction

Malignant gliomas (MG) are highly aggressive primary brain cancers with a very rapidly progressing clinical course despite aggressive treatment with best available treatment strategy [1]. Median survival is only 20.9 months with 5-year survival of only 5–10% [2,3]. While the scientific community is working diligently toward a scientific breakthrough that will alter the clinical course of this disease, it is of equal importance to help patients suffering from this disease maintain a good health-related quality of life (HRQoL). Unlike end of life hospice care, palliative care is comprehensive treatment to ease the discomfort, symptoms and stress of serious life-threatening illness, such as cancer that can continue along with life prolonging treatments and follow-up [4,5]. Integration of palliative care along with conventional oncological treatment has shown to significantly increase the quality of life of cancer patients [6,7]. Over the last decade, HRQoL has increasingly become an integral part of mainstream comprehensive treatment strategy for oncological care [8,9,10,11], which is reflected by the fact that almost 10% of all randomized cancer clinical trials include HRQoL as one of the primary end points [12]. In addition, The US Food and Drug Administration takes the HRQoL benefit of any new anticancer drug into consideration for clinical approval. 

HRQoL is a complex concept that has been historically hard to define in a standard manner; as such, it has been defined in many different ways in the literature with an overall common concept that encompasses the multidimensional wellbeing of a person in terms of their overall satisfaction with life [13,14,15,16]. Due to the subjective nature of this domain, it has been hard to objectively measure this in a standardized manner across all patients, as well as interpret results from various studies and translate the findings into busy clinical practice [17,18,19,20]. 

Over the last several years, use of patient-reported outcome (PRO) questionnaires has emerged as a standard practice in the assessment of cancer patient HRQoL [13,21,22]. PROs provide an assessment of a patient’s wellbeing that comes directly from the patient without the interpretation of the patient’s responses by a physician or other practitioner. It also incorporates disease symptoms description, patient functioning, and quality of life questionnaires [23,24,25]. Some data that can only be obtained from patients using PROs are: symptoms (such as headache, fatigue, anxiety, depression etc.), frequency and severity of the symptoms, and how the symptoms or the disease effect patients’ daily life etc. [21,26,27].

Patients with brain tumors face serious and unique challenges with neurologic and neuropsychological problems that are specific to the location of the tumor and just not the systemic symptoms of the cancer. Brain tumor patients score significantly lower in all domains of functioning compared to age-matched and sex-matched healthy controls and have lower social functioning and more problems with vision, motor functions and communication, compared to patients suffering from other cancers [28,29]. Current methodologies being used in the clinical setting for brain tumors involve HRQoL questionnaires, proxy-rated HRQoL measures, and measures of instrumental activities of daily living [30]. In addition to focal neurologic deterioration, brain tumor patients face several unique challenges that make reporting of outcomes difficult. These include motor deficits, personality changes, cognitive deficits, aphasia, and visual field defects [31,32]. Because of these neurological deficits, the use of standardized questionnaires may not be a reliable way to measure HRQoL in this particular group of patients. Meanwhile, studies analyzing the accuracy of caregivers to provide reliable proxy HRQoL ratings showed that for cognitively impaired patients, caregivers do not report accurate HRQoL ratings [33]. In addition, various studies have shown that a large proportion of brain tumor patients suffer from mood disturbances such as anxiety and depression [31,34,35,36]. More importantly, in some cohorts of primary brain tumor patients, depression was the most important independent predictor of quality of life and was found to have an adverse impact on survival [37,38,39]. Thus, previous research indicates there is an unmet need for a better tool to asses HRQoL in brain tumor patients; one that does not rely solely on the patient’s ability to communicate and interact clearly or depend on their caregiver’s proxy ratings.

Animal-assisted therapy is a growing field that typically utilizes dogs trained to be obedient, calm, and comforting to help people recover from or better cope with health problems such as cancer, mental illness, etc. Several studies have reported significant pain relief after participating in therapy dog visits. Objective reports of reduced pain and pain-related symptoms are supported by studies measuring decreased catecholamines and increased endorphins in humans receiving friendly dog visits [40,41,42]. There is a growing body of literature that shows that animals can reduce tension and improve mood. Specifically in the setting of depression, along with traditional treatment modalities, pets can help people with mild to moderate depression feel better [43]. Pet animals have been used in a multitude of medical scenarios, and several recent studies have shown that animal-assisted therapy can significantly reduce pain, anxiety, depression and fatigue in cancer patients [44,45,46]. Animal-assisted therapies that mostly involve interaction with trained therapy dogs have been used in adult and pediatric acute care hospital settings as well as in outpatient clinics, nursing homes and rehabilitation centers [41,47,48,49,50].

In this paper, we describe our unique study that was designed to address two critical questions: (1) can pet therapy in the outpatient setting help improve HRQoL of brain tumor patients? and (2) can patient’s facial expression be used as a proxy measure for their overall HRQoL? The framework of this study originated from the desire to explore and combine non-conventional modalities to overcome the limitations of conventional methodologies. Since facial expressions are best captured by an artist’s lens, we combined pet therapy along with an artist’s involvement to address the questions. Use of facial expression to measure patient’s wellbeing has never been tested in any clinical setting. Moreover, there have never been any studies conducted using this approach of combining pet therapy and study of facial expression. We believe that collaboration between clinical research, art and related therapies could be symbiotic and produce useful scientific as well as artistic outcomes. 

The scientific goal of the study was to see if a short pet therapy session could improve a patient’s quality of life, as determined by their change in responses to the standard quality of life questionnaire (QOL-C30/BN20) before and after the session. In addition, still photographs were taken before and after the pet therapy session to assess if facial expression could be used as a surrogate to measure or infer HRQoL.

The artistic goal of the study involved taking excerpts of audio interviews and slow-motion video clips captured of the patients and creating a series of 2–6 min video portraits that were exhibited and shared with the public to tell the stories of patients affected by brain cancer. The video portraits served as a unique platform to showcase how it feels to face a lethal disease head on, and in doing so, remind the public to value the everyday opportunities and joys which life brings. 

One unexpected positive outcome that emerged early on from this study was the observation that patients having the opportunity to share their experience of living through a life altering disease with a non-medical person improved their quality of life. As reported by the patients themselves, the interaction with the artist through audio interviews and video portraiture, significantly enhanced patient’s quality of life. The approach of engaging an audience or a patient directly into the art-making process is referred to as participatory art. Over recent years, there is evidence that points to the health benefits associated with participatory arts [51]. Involvement in participatory art in the form of expressive writing, music, dancing etc. not only improves overall wellbeing of patients, it also has positive impacts on specific health parameters such as depression, anxiety and immune system functioning [9,52,53,54]. 

By incorporating interactive non-traditional therapy and artistic sessions with patients, the study sought to investigate if the integration of clinical care, pet therapy and art-making could positively impact the quality of life of brain tumor patients. Thus, the study describes a novel way to incorporate unconventional therapies into overall patient care without disrupting the busy daily clinical workflow. 

## 2. Methods

**Figure 1:**
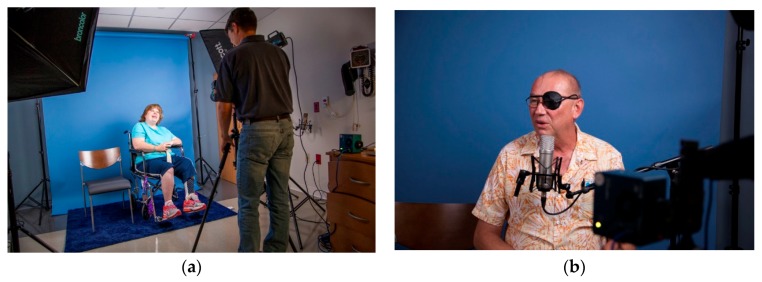
Typical patient study room used for project. (**a**) artist speaking with a patient during the photography phase of the session; (**b**) Patient responding to prompt to describe how his outlook on life changed because of his brain tumor diagnosis.

The clinical research nurse approached brain tumor patients, who meet the inclusion criterion of the study, for study consent. Patients who consented to the study were invited to participate in a study session. Upon arrival at the study session, patients were asked to complete the standardized QOL-C30/BN20 questionnaire. Patients were then led into the studio room and met with the artist who introduced the goals of the project and took a series of still photographs and slow-motion videos for a few minutes (Figure 1). Patients were then asked a few questions about how their day-to-day life has changed with their diagnosis. Their responses were audio recorded. Following the audio/video session, the clinical trial nurse introduced the patient to the therapy dog. After initial introduction, the patient is left alone to interact with the therapy dog for 10–20 min. This session is video recorded. Upon completion of the pet therapy session, the therapy dog was led out of the studio, and the patient was asked to complete the standardized QOL-C30/BN20 questionnaire again. Immediately afterward, the patient was again photographed/videotaped and then asked to respond to a few questions about how their perspective on life changed with their diagnosis.

The study is currently ongoing with an initial goal to accrue 50 patients. In this paper, we describe our findings from first 9 patients enrolled.

## 3. Results

### 3.1. Impact of Pet Therapy on Patients

From our initial observation, we noticed that patients were very eager to meet the therapy dog and anticipated her arrival very enthusiastically. Patients spent most of the pet therapy session physically interacting with the dog by hugging, petting, and playing with her. One of the consistent messages conveyed by most patients in the study was feeling as though they are their disease rather than an actual person. By participating in these study sessions, it allows the patient to tell their story to an unbiased audience and feel as though they are being heard. In addition, having the non-judgmental calming presence of the unassuming therapy dog, although transiently, provided them a friend who is just there for them without any questions or solution. 

**Figure 2:**
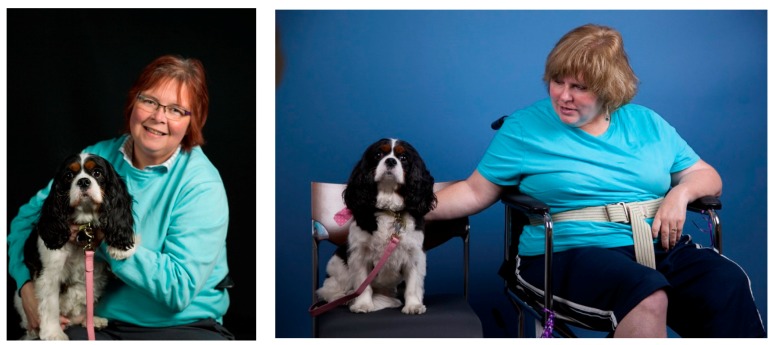
Patient interactions with Pet Therapy dog: Cleopatra Dey, a Cavalier King Charles Spaniel.

When the therapy dog entered the room, most patients met her with a smile on their face. The patients immediately looked to the dog and began interacting with her as soon as she got to her seat. There was a palpable shift in the mood of the room once the therapy dog was introduced. Patients appeared to relax and were excited to have time to just sit and pet the dog without interruption (Figure 2).

The pet therapy component of the session was the only time the patients had to be alone in the room. This allowed the patient time to reflect on the session and have some quiet time to him or herself while petting the therapy dog. Not having to converse with another person seemed to enhance the therapeutic effect of the pet therapy session. When the artist and study nurse re-entered the room, patients appeared more at ease and this observation by our team was confirmed by verbal feedback from the patients. We also captured the change formally using QOL-C30/BN20 questionnaire that will be formally analyzed together for all the patients at the end of the study. Preliminary data analysis from the nine patients showed significant improvement in long-term outlook on life after the session.

### 3.2. Impact of the Portrait Session on Patients

Having an artist interact with patients to record how their diagnosis has affected them appeared to offer another promising way to improve quality of life outcomes in patients facing difficult diagnoses. The artistic intervention employed in this study offered a platform for the patient to share their story with others, giving the patient the unusual role of being the expert in a health care setting. In clinical care settings, patients typically play a psychologically passive role, expecting that the physician or medical professional will take the lead in caring for him or her. When a doctor asks how a patient is doing, they mostly think in terms of physical health, not their emotional health. Even when a patient works with a clinical psychologist, the patient assumes they are there to be fixed. 

However, in our study setting, an artist who assumed the role of a neutral party rather than a clinician greeted the patient in a studio-like setting. From the beginning, the patient was treated like the expert and told of the importance and value of sharing their story so that the larger community can understand how it feels to face being diagnosed with such a daunting situation. 

Both the artist and pet therapy sessions occurred in a calm setting to promote an environment that encourages reflection on their disease. We did not want the interview session to become a photo shoot, where the patient just becomes a stand-in for a model. We did not try to make them look “good” or fashionable. Rather, the goal of our study was to make them look like themselves and let their personality come through. The same approach was taken when making audio recordings of patients talking about how their diagnosis has changed their routine and outlook on life. This approach kept the emphasis on the patient and ensured that the patient felt his or her voice was highly valued. Thus, the artist session really elevated the importance of how the patient’s diagnosis has impacted their life, and how they find meaning in life post-diagnosis. 

Anecdotally, patients often told the nurse at the end of the session or the neurosurgeon at their next clinical checkup how much they enjoyed participating in the study and had a chance to share their story with the artist. This may be because rather than focusing on just their condition and deficits, they saw participation in the study as being part of a larger effort, doing something for others instead of just themselves. Often just focusing on the self is difficult for brain tumor patients, either because it brings up fears and anxiety about their diagnosis, or they already feel too much at the center of attention. Patients who are diagnosed with a difficult illness can also feel disempowered by their situation. They can feel fatalistic and depressed by their lack of control. This study’s artistic intervention, where the patient becomes central to the creation of something with implied social value and relevance (in this case an artwork that can be shared with the public), can help them feel more empowered as a person. While the creation of a public art project may not be a scalable model for enhancing patient wellbeing for brain tumor patients, the premise of finding a way to make a patient’s experience and insight valued and useful likely is. 

**Figure 3:**
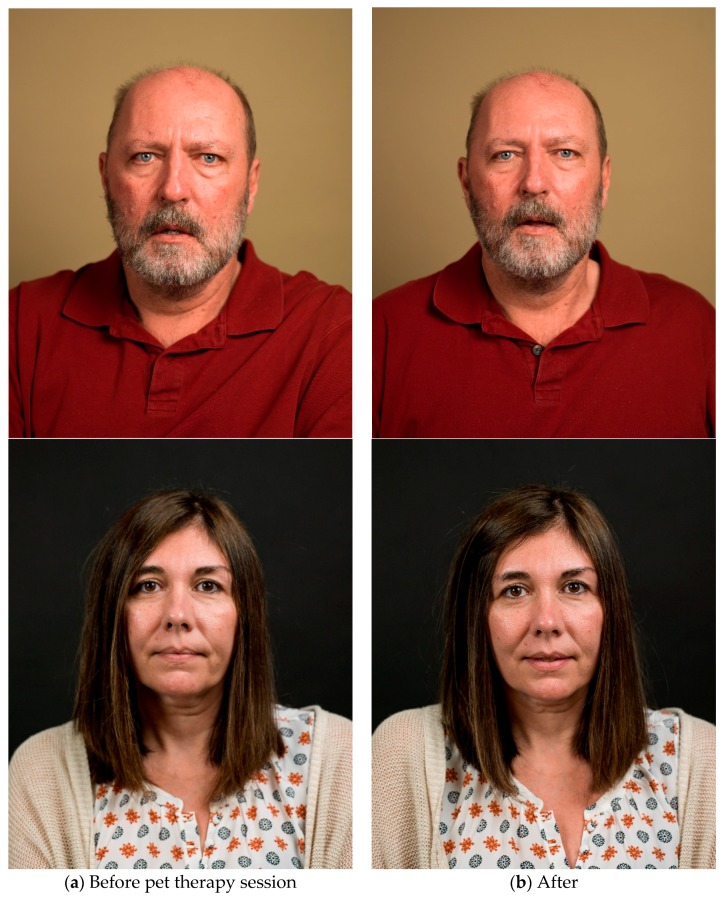
Examples of the photographs taken (**a**) before the pet therapy session and (**b**) after the pet therapy session.

Early study results suggested there is significant value in the personal and non-traditional engagement with patients outside of standard medical treatment. Especially, the artist who interacted considerably with the patient felt this change in the patient’s outlook over the course of the patient session. At first, patients were generally more reserved and waited for direction from the artist. By the end of the session, when the second round of photographs, video, and audio recordings were taken, patients appeared more relaxed, less reserved, and more open to speaking about their condition and their life, clearly demonstrating the therapeutic benefit of the pet therapy session. In addition, patients seemed more positive and smiled more. All seemed genuinely engaged and glad to have participated in the study. This change seemed apparent when looking at many of the photographs taken at the beginning of the session versus those taken later, after the pet therapy component (Figure 3).

When asked informally about their experience of participating in the session, patients expressed positive feedback saying things such as: “I feel calmer. I do think there is a soothing aspect to it” and “It brings a sense of calm in an environment full of scans, medicines, and charts.”

### 3.3. Impact of the Study on the Community

**Figure 4:**
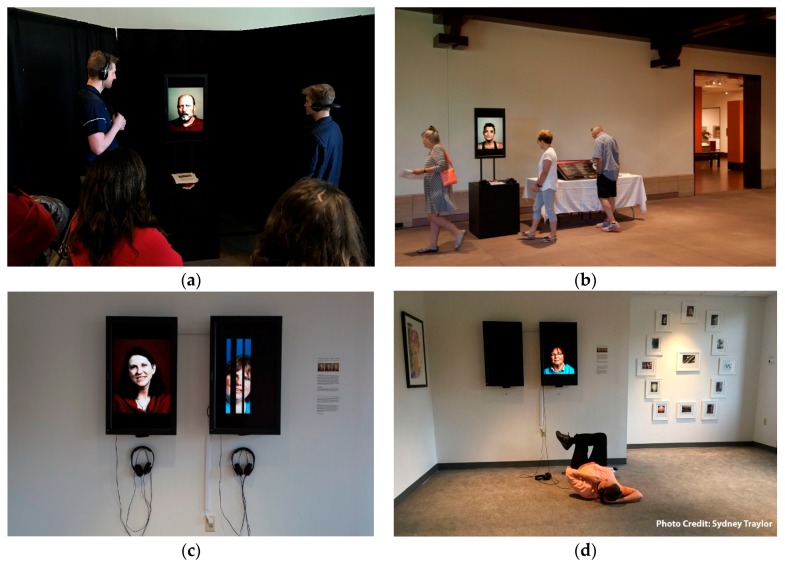
Public exhibition of video portraits made from artist recordings with patients. (**a**) 21st Century Great Conversations in Neuroscience, Art, and Related Therapeutics Symposium, IUPUI. April 2017; (**b**) Study Presentation at Eiteljorg Museum, Indianapolis, IN in June 2017. Both a poster about the study, including details about pet therapy aspect and select video portraits made from the artist were on display; (**c**,**d**) Vitality Through Art Exhibition, Marian University, Indianapolis, IN, November 2017. Public Exhibition of video portraits made from artist recordings with patients.

In this one of a kind study, that spans the boundaries of art and science, we were pleasantly surprised by many unexpected positive outcomes that were not initially accounted for. The study not only had a direct beneficial effect on the patients enrolled in the study, it also appeared to have a significant impact on the community by way of the art created during these sessions. The artist engagement component of the study, including patient interviews and filming, resulted in the creation of individual video portraits of each patient. The videos utilize slow motion video and audio of patients telling the story from diagnosis to how their outlook on life has changed. To date, these videos have been exhibited three times in the Indianapolis area, including at the 21st Century Great Conversations in Neuroscience, Art and Related Therapeutics Symposium that took place on the IUPUI campus in April 2017 (Figure 4). These videos are also accessible online through the portal: www.braintumordiaries.org. The website not only allows the public open access to listen to the patients’ stories, but it allows participants and their families easier access as well. The participants and their families have responded very positively to the videos. For those families that have unfortunately lost a family member to brain cancer, it has become even more valuable. 

The Public has responded very positively to these artistic exhibitions of brain tumor patients, describing them as “meaningful” and “important” while acknowledging the difficulty in listening to a person who has been dealt such a difficult reality. Individuals who stop and listen to these patients’ stories come away touched and from an artistic perspective. That was precisely the goal—to offer people an opportunity to recognize that life is fleeting, even fragile and unfair, but the resilience of the human spirit, even in the hardest moments is strong. So, although the subject is difficult and bleak, these portraits are meant to serve as an inspiration to the rest of us about how even when compromised by illness, there is much to be had in the human experience. We will be monitoring this trend of positive impact on the community by tracking the number of visits to our website and by counting the number of invitations received from the community to present our work in public settings.

## 4. Discussion

This unique project began when two of the authors engaged in a discussion about the cross section of art and science, and how the two might collaborate to study how brain tumor patients’ quality of life could be improved by engagement in non-traditional experiences outside of standard clinical care. The current study was born out of the belief that collaboration between art and science could be symbiotic and produce useful outcomes on multiple fronts. 

The one of a kind nature of the project also created several challenges that we had to work though. The use of a therapy dog is an integral part of this study, but it also introduces an inherent “dog lover” bias to the study. All the patients who consent to the study are self-proclaimed “dog lovers”, and they appear to be excited by just the prospect of enrolling in the study. While use of animals to decrease anxiety and improve mood have been documented in other health settings, it remains underemployed as a tool to improve patient wellbeing in standard clinical practice. In our study, the pet therapy session happens in the outpatient clinical setting. Since brain tumor patients sometimes spend significant time in the hospital, the feasibility of this method needs to be tested in inpatient settings.

The methodologies employed between science and art are vastly different and often appear incompatible. This created a healthy challenge for us as we approached a study design that would give adequate space for both an artist and scientific researcher to feel that their efforts could be shaped into meaningful outcomes in their respective disciplines. On one hand scientific methodologies such as QOL-C30/BN20 questionnaire are very objective; whereas, on the other hand, the experimental methodology, the analysis of facial expression is subjective. In the ideal setting, use of multimodal tools that combines PRO along with analysis of facial expression will provide a more comprehensive assessment of HRQoL in brain tumor patients. 

Since there have been no previous studies to provide us a pre-established framework, we had to leave some fluidity in our study structure. The initial goal of our study was to study the effect of pet therapy, and we did not anticipate the positive effect of participatory art that arose from the patients’ interactions with the artist. Thus, our current study is not designed to delineate the effect of pet therapy vs. participatory art-making. 

Another significant aspect of the study was the involvement of the artist in the study. It may be difficult to arrange close collaboration between artists and scientists except in larger academic or urban settings where both medical researchers and artists can easily interact. Furthermore, larger universities may allow for unique grant mechanisms that encourage transdisciplinary research to foster such research between distinct disciplines and fields of research. 

Even with these limitations, when it comes to the subject of trying to help people and improve quality of life, science and art are often working toward similar goals, and we were able to design a working protocol that is flexible enough to allow for the analytical needs of research as well as the creative freedom essential for the creation of art. Our goal in publishing the early findings of this study is to inspire more people to engage in designing and executing studies that blur the line between very dissimilar fields and establish innovative tools to improve the quality of life of patients. 

The interactive session employed by this study had a clear impact on the patients who have so far participated, and we anticipate the trend of significant improvement in a patient’s long-term outlook on life will continue as the study pool grows. Due to the limitation of the study design, we cannot say with certainty if this change is due to the intervention with the artist or the pet therapy session or both. Future studies must be designed to accommodate two arms, one studying the role of pet therapy and another the role of participatory art-making separately. In addition, feasibility of the study in the inpatient clinical setting will also need to be evaluated. Based on the feedback we received from the community engagement in the project, one way to fund such creative study designs in the future might be by public exhibition of the artistic works produced. 

In addition, patient enrollment has been harder than expected, partially due to the impact of brain tumors on patient independence. The fact that most brain tumor patients cannot drive themselves to the clinic, and the understanding that many patients live more than an hour away from where the study is conducted makes it unfeasible for many to participate. 

In conclusion, it is of paramount importance for medical practitioners to consider the whole person when they are treating a patient. Patients often feel high levels of stress and anxiety around their diagnosis and while practitioners are generally sensitive and compassionate to this, alternative non-traditional interventions such as pet therapy and artist led projects, as well as other engagement approaches should be considered in the context of the patient’s HRQoL. This study serves as a model for how artists and scientists can work together to improve patient’s experiences and create valuable research findings and artistic output concurrently.
